# Skipping the Drip: Intravenous Proton Pump Inhibitor Bolus Therapy Leads to Poor Outcomes in High-Risk Bleeding

**DOI:** 10.7759/cureus.8362

**Published:** 2020-05-30

**Authors:** Raja Samir Khan, Yousaf B Hadi, Noor Chima, Justin Kupec

**Affiliations:** 1 Internal Medicine, West Virginia University School of Medicine, Morgantown, USA; 2 Internal Medicine, J.W. Ruby Memorial Hospital, Morgantown, USA; 3 Medicine, West Virginia University School of Medicine, Morgantown, USA; 4 Gastroenterology and Hepatology, West Virginia University School of Medicine, Morgantown, USA

**Keywords:** peptic ulcer, upper gastrointestinal bleeding, proton pump inhibitor

## Abstract

Background and Aim

The aim of this study was to evaluate the impact of a change in our institute’s protocol from continuous intravenous (IV) proton pump inhibitor (PPI) therapy to bolus IV PPI therapy for the treatment of peptic ulcer-related bleeding on patient outcomes. Current guidelines recommend PPI therapy through high-dose IV bolus followed by continuous infusion for bleeding ulcers. Conflicting data have been reported regarding the practice shift to intermittent IV PPI therapy.

Methods

A retrospective record review was conducted of patients treated at West Virginia University between 2017 and 2018 for peptic ulcer related bleeding who underwent endoscopy and had high-risk stigmata. Relevant variables were identified. Outcomes were compared between groups based on PPI strategy. The primary endpoint was any poor outcome defined as rebleeding, need for embolization or surgery, or mortality during hospital stay.

Results

A total of 130 patients were included, with a mean age of 62.18 years. Continuous PPI infusion was used in 39.23%, whereas bolus IV PPI was used 60.76%. Poor outcome was encountered in 11 (21.57%) patients in the continuous and 33 (41.77%) patients in the bolus group (p = 0.028). On multivariable analyses, bolus PPI strategy was independently linked to poor outcome (Wald’s odds ratio: 2.8; 95% CI: 1.21-6.84; p = 0.019) and an increased need for embolization/surgery (OR: 4.12, 95% CI: 1.14-19.99; p = 0.046).

Conclusions

IV bolus therapy showed worse outcomes compared with continuous IV PPI therapy for patients with peptic ulcer bleeding with high-risk features. More robust data are needed before a practice shift to bolus PPI may be appropriate.

## Introduction

Peptic ulcer disease (PUD) remains one of the most common causes of upper gastrointestinal (GI) bleeding [1]. It has been well documented in the literature that upper GI bleeding secondary to PUD carries a mortality rate of more than 2% along with a financial burden of greater than seven billion USD annually [2]. Current guidelines recommend proton pump inhibitor (PPI) therapy in the form of an intravenous (IV) bolus followed by a continuous infusion after endoscopic treatment in patients with bleeding ulcers with high-risk endoscopic findings (active bleeding, non-bleeding visible vessels, and adherent clots) [3,4]. When given intravenously and at a high dose, PPIs have been documented to maintain a neutral gastric pH [5]. High-dose PPI regimens have been shown to reduce both mortality and rebleeding in patients with peptic ulcer related bleeding with high-risk features [4]. Conflicting data, however, suggest substituting intermittent IV PPI therapy for the currently recommended therapy of bolus plus continuous infusion, with recent studies supporting its non-inferiority [6]. In the current climate of healthcare driven by patient safety, quality, and cost reduction, there are limited data on cost-effectiveness and resource utilization relative to the two administration routes. We conducted a retrospective cohort study to evaluate the impact of a recent shift in institutional protocol from continuous infusion PPI therapy to bolus therapy in peptic ulcer related bleeding.

## Materials and methods

Study design and population

A retrospective cohort study of all patients who presented with peptic ulcer related bleeding at West Virginia University Medicine was conducted. The study was reviewed and approved by the Institutional Review Board prior to initiation. All patients with upper GI bleeding who underwent esophagogastroduodenoscopy (EGD) at our institution between 2017 and 2018 were identified, and their medical charts were reviewed. Patients older than 18 years of age were included if (a) they were diagnosed with peptic ulcer related bleeding, (b) an EGD was conducted within 24 hours of admission, and (c) EGD findings revealed high-risk stigmata (defined as Forrest class 1a, 1b, 2a, or 2b ulcers).

Study data

Medical charts were reviewed to extract demographic and clinical variables of interest, including age, gender, smoking history, medication lists, co-morbidities, findings on EGD, and clinical outcomes including rebleeding, inpatient mortality, length of stay, need for transfusion, and need for embolization or surgery.

PPI therapy regimens

Patients were divided into two groups on the basis of treatment with continuous or bolus IV PPI regimens. After the administration of a one-time bolus of 80 mg IV pantoprazole, the continuous PPI regimen at our institution is comprised of continuous pantoprazole for 16 hours per day for three days. This is transitioned to oral PPI if bleeding stops and alimentation is possible. Bolus regimen is comprised of IV pantoprazole 40 mg twice daily for three days with transition to oral PPI if bleeding stopped and the patient was suitable for alimentation.

Study outcomes

The primary endpoint of the study was any poor outcome. Poor outcome was defined as rebleeding, need for embolization or surgery, or mortality during hospital stay. Secondary endpoints were the occurrence of rebleeding, need for embolization or surgery, need for transfusions, and mortality during hospital stay.

Statistical analyses

Bivariate analyses were conducted using Student’s t tests, Fisher’s exact tests, and chi-square tests for continuous and categorical variables. P-values less than 0.05 were considered significant for the purposes of this study. A multivariable logistic regression model was fitted to assess independent associations and control for confounding variables. Separate multivariable logistic regression models were fitted for outcomes including rebleeding, inpatient mortality, length of stay, and need for embolization or surgery. The R statistical software (R Foundation for Statistical Computing, Vienna, Austria) was used to conduct all analyses.

## Results

Patient characteristics

A total of 130 patients with high-risk ulcers were included. The sample comprised a majority of male participants (60.0%), and the mean age of the study population was 62.18 years (standard deviation [SD]: 17.63). At the time of hospitalization, 45.38% of the population endorsed a history of smoking, and 31 patients (23.85%) were using non-steroidal anti-inflammatory drugs (NSAIDs) at home. Baseline characteristics of the population are summarized in Table 1.

**Table 1:** Baseline characteristics of the study population. PPI, proton pump inhibitor; SD, standard deviation; NSAID, non-steroidal anti-inflammatory drugs

Variable	Continuous PPI group	Bolus PPI group	p-Value
Age in years, mean (SD)	64.59 (SD: 14.52)	60.62 (SD: 19.21)	0.18
Male gender	28 (54.90%)	50 (63.29%)	0.19
Medication use at home			
Antiplatelets	22 (43.14%)	33 (41.77%)	0.44
Anticoagulants	13 (25.49%)	12 (15.19%)	0.22
NSAIDs	15 (29.41%)	16 (20.25%)	0.32
Smoking history	26 (50.98%)	33 (41.77%)	0.39
Ulcer location(s)			
Gastric	36 (70.59%)	47 (59.49%)	0.69
Duodenal	22 (43.14%)	42 (53.16%)	0.52
Ulcer grade			0.24
Forrest class 1A	1 (1.96%)	5 (6.33%)	
Forrest class 1B	19 (37.25%)	27 (34.18%)	
Forrest class 2A	16 (31.37%)	24 (30.38%)	
Forrest class 2B	15 (29.41%)	23 (29.11%)	

Continuous PPI infusion was used for 51 (39.23%) patients, whereas the rest of the patients (60.76%) received an intermittent bolus IV PPI regimen.

Outcomes

The composite poor outcome, as defined in the study methods, occurred in 44 (29.0%) patients in the study population. Univariable analyses revealed that age, gender, smoking history, anticoagulant or antiplatelet medication, and NSAID use were not associated with poor outcome. Poor outcome was encountered in 11 (21.57%) patients in the continuous and 33 (41.77%) patients in the bolus group, (chi-square p-value = 0.028). On multivariable analyses, PPI strategy was independently linked to poor outcome (Wald’s odds ratio: 2.8; 95% CI: 1.21-6.84; p = 0.019). Associations of variables with poor outcome are detailed in Table 2.

**Table 2:** Association of baseline variables with poor outcome on bivariate and multivariate analyses (p-values). NSAID, non-steroidal anti-inflammatory drug; PPI, proton pump inhibitor

Variable	Bivariate analyses	Multivariable analyses
Age	0.92	0.60
Male gender	0.42	0.77
Smoking	0.84	0.58
NSAID use	0.39	0.30
Antiplatelet use	0.96	0.84
Anticoagulant use	0.16	0.14
Need for endoscopic hemostatic therapy	0.28	0.08
Bolus PPI regimen	0.028	0.019

On univariable analysis, rebleeding rates, need for transfusion, and mortality were not statistically significant in our study population, irrespective of PPI regimen (p-values are detailed in Table 3).

Separate multivariable binomial logistic regression models were fitted for all secondary outcomes, with age, gender, smoking history, NSAID use, antiplatelet use, anticoagulants, need for endoscopic hemostatic therapy, and type of PPI regimen (bolus or continuous) as covariates. Bolus PPI regimen was independently associated with an increased need for embolization/surgery (OR: 4.12; 95% CI 1.14-19.99; p = 0.046). The need for endoscopic hemostatic therapy was independently associated with rebleeding (p = 0.048), need for transfusion (p = 0.037), and embolization/ surgery (p = 0.018) during hospital stay (Table 3).

**Table 3:** Outcomes in the continuous and bolus PPI groups. PPI, proton pump inhibitor; SD, standard deviation

Variable	Bolus PPI	Continuous PPI	Univariable p- value
Rebleeding	16 (22.22%)	9 (15.25%)	0.18
Poor outcome	33 (41.77%)	11 (21.57%)	0.028
Mortality	11 (13.92%)	4 (7.84%)	0.44
Need for embolization/surgery	14 (17.72%)	3 (5.88%)	0.09
Need for transfusion	64 (81.01%)	43 (84.31%)	0.81
Mean hospital stay (days)	11.96 (SD: 13.02)	10.02 (SD: 17.37)	0.55
Readmission	7 (8.86%)	2 (3.92%)	0.47

## Discussion

Our data, which is from a tertiary care center experience, notes worse outcomes with IV bolus PPI therapy compared with continuous infusion for acid suppression therapy in the management of peptic ulcer related upper GI bleeds. These findings are in contrast to the findings of some previously published studies on this subject. A systematic review and meta-analysis of all 13 published and unpublished studies comparing IV bolus PPI regimens with continuous PPI therapy in patients with bleeding ulcers and high-risk endoscopic findings showed no significant increase in rebleeding within 3, 7, and 30 days, mortality, urgent interventions, blood transfusions, and hospital length of stay [6]. Only one of these studies was conducted in a western Caucasian population, with the rest from primarily Asian populations, and differences in ethnicities and racial characteristics may affect the generalizability of their findings and may explain the difference in results that we have experienced in our institute [7]. Furthermore, the studies included in the systematic review had heterogenous bolus PPI regimens, and therefore definitive conclusions cannot be drawn about the non-inferiority of any single bolus PPI regimen.

The recent interest in bolus PPI regimens is based on the ease of administration and potential reduction in healthcare resource use. Many institutions, including ours, have transitioned from PPI infusions to bolus therapy since recent evidence supports the non-inferiority of bolus PPI regimens. A budget impact analysis noted that the incremental costs of using continuous PPI regimen compared with bolus PPI regimens are only modest when compared with total costs associated with PUD bleeding related admissions [8-10]. Per an analysis using a decision model from U.S. third-party payers’ perspective, compared with the mean cost per patient for admission with continuous PPI regimens of 11,293 USD (95% CI: 11,215-11,374), shifting to intermittent IV PPI would result in a charge of 11,208 USD (95% CI: 11,096-11,253) [11]. A practice shift to bolus PPI may not be feasible in terms of quality or cost reduction until more evidence supports the use of a bolus IV PPI regimen to reduce mortality and rebleeding [12,13]. The latest guideline by the International Consensus Group has not recommended bolus PPI therapy for peptic ulcer related bleeding and still favors IV continuous high-dose infusions with an initial 80 mg IV bolus followed by 8 mg/kg for 72 hours [4]. The consensus group was not confident that the precision of the estimates of the absolute differences between these regimens regarding mortality and rebleeding is sufficient to warrant their recommendation. Overall, it appears that there is still a lack of consensus regarding the transition to bolus PPI regimens compared with continuous high-dose PPI regimens. Of note, large heterogeneity exists in terms of both endoscopic therapy and dosing of PPI in the studies available, which have been analyzed in the systematic review by Sachar et al. and reviewed by the International Consensus Group [4,6]. The systematic review has included studies that have used monotherapy for hemostasis, which is no longer the standard of care.

The strengths of our study include the administration of uniform continuous PPI and IV bolus regimens in all patients in their respective groups. As IV PPI is administered by an electronic medical record driven order set, all patients received the assigned regimen for 72 hours. We included only the high-risk ulcer group in this analysis, who have a guideline-based indication for IV PPI regimen. This is also the first real-world data from a primarily Caucasian population on this subject. However, our study is limited by its single-center nature. Furthermore, we have only studied the 40-mg IV twice daily regimen of bolus IV therapy in our analysis, which is the most commonly used regimen in previous trials comparing bolus and continuous regimens, and thus conclusions cannot be drawn regarding other IV bolus regimens from our data [6].

## Conclusions

In conclusion, in our real-world retrospective cohort analysis, we found worse outcomes with IV bolus PPI therapy when compared with continuous infusions of PPI for patients with peptic ulcer bleeding with high-risk features. Our study reaffirms the conclusions drawn by the International Consensus Group regarding the insufficient evidence of non-inferiority of bolus regimens for this group of patients. There remains a need for more robust data and better studied bolus PPI regimens before a practice shift may be appropriate.
